# Genotype Distribution and Prevalence of Human Papillomavirus in Head and Neck Cancer Samples from Istanbul, Turkey

**DOI:** 10.3390/pathogens10121533

**Published:** 2021-11-23

**Authors:** Muammer Osman Köksal, Başak Keskin Yalçın, Fahriye Keskin, Sevgi Çiftçi, Ibrahim Yağcı, Seyhan Özakkoyunlu Hasçiçek, Bora Başaran, Kemal Değer, Ali Ağaçfidan, Alexander Quaas, Baki Akgül

**Affiliations:** 1 Department of Medical Microbiology, Istanbul Faculty of Medicine, Istanbul University, 34093 Istanbul, Turkey; muammerosmankoksal@istanbul.edu.tr (M.O.K.); aagacfidan@hotmail.com (A.A.); 2 Department of Oral and Maxillofacial Surgery, Faculty of Dentistry, Istanbul University, 34116 Istanbul, Turkey; basakkeskin86@hotmail.com; 3 Unit of Microbiology, Istanbul Faculty of Dentistry, Istanbul University, 34116 Istanbul, Turkey; sevgiciftci@gmail.com; 4 Department of Otorhinolaryngology & Head and Neck Surgery, Sisli Hamidiye Training and Research Hospital, University of Health and Science, 34371 Istanbul, Turkey; ibrahimyagcimd@gmail.com; 5 Department of Pathology, Sisli Hamidiye Training and Research Hospital, University of Health and Science, 34371 Istanbul, Turkey; shascicek@gmail.com; 6 Department of Otorhinolaryngology & Head and Neck Surgery, Istanbul Faculty of Medicine, Istanbul University, 34093 Istanbul, Turkey; bbasaran@istanbul.edu.tr (B.B.); kdeger@istanbul.edu.tr (K.D.); 7 Institute of Pathology, University Hospital Cologne, 50937 Cologne, Germany; alexander.quaas@uk-koeln.de; 8 Institute of Virology, University of Cologne, Faculty of Medicine and University Hospital of Cologne, 50935 Cologne, Germany; baki.akguel@uk-koeln.de

**Keywords:** head and neck squamous cell carcinoma (HNSCC), human papillomavirus (HPV), oral cavity, oropharyngeal squamous cell carcinoma (OPSCC), p16INK4A

## Abstract

Human papillomavirus (HPV)-associated tumors account for a significant proportion of head and neck squamous cell carcinomas (HNSCC) in developed countries. In recent years, there has been a rise of HPV infections associated with HNSCC, especially HPV16, which is the most commonly detected type in oral and oropharyngeal cancers. To investigate the frequency of HPV-driven HNSCC among patients living in Turkey, HPV DNA positivity and p16INK4A expression were assessed in primary tumor biopsies (n = 106). Eighteen out of one hundred and six (19%) HNSCC tumors showed p16INK4A overexpression, and 26/106 cases (24.5%) were positive for HPV DNA. Sixteen out of twenty-six samples were positive for both HPV DNA and p16INK4A staining. HPV16 could be isolated from 22/26 samples (84.6%) and was found to be the most frequently detected HPV type. This study represents the largest cohort of Turkish patients with HNSCC characterized according to HPV status and p16INK4A expression. Our data suggest that HPV16 infection, along with smoking, contribute to the development of HNSCC.

## 1. Introduction

Squamous cell carcinoma of the head and neck (HNSCC) are anatomically heterogeneous neoplasms originating from mucosal surfaces of the oral cavity, oropharynx, hypopharynx, larynx, and nasopharynx. Approximately 263,000 cases of oral cavity cancer and 135,000 cases of pharyngeal cancer are reported worldwide each year [1]. Head and neck cancer is the sixth most prevalent type of cancer, and there are profound differences in the incidence rates of HNSCC among geographic regions [2]. In general, the number of cancer cases is higher in North America (60.4%) than in Europe. Within Europe, HNSCC frequency appears to be higher in northern countries (56.5%) than in southern countries (24.2%) [3]. Over the past decade, scientists across the globe have observed a steady increase in oropharyngeal squamous cell carcinoma (OPSCC) and a decrease in cancers of the larynx and hypopharynx [4]. OPSCC is diagnosed four times more frequently in males than females in the US [5]. In addition, it is more frequently observed in individuals over the age of 60 [4]. Tobacco and alcohol consumption are among the most important risk factors in the etiology of this cancer type [6]. However, since the 1980s, numerous studies have shown that the human papillomavirus (HPV) can contribute to the etiology of HNSCC types [1,6]. Worldwide, HPV-related OPSCCs increased from 7.2% in the 1990s to 32.7% in the 2010s, possibly as a result of changes in sexual behavior [7]. The number of HNSCC diagnoses related to intensive abuse of tobacco and alcohol in the elderly is globally decreasing as a result of the overall decline in tobacco use [8]. By contrast, cases of HPV-related OPSCC mainly induced by HPV16 are on the rise, predominantly among young people in North America and Northern Europe [8,9]. This cancer type is associated with HPV infection as well as sexual behavior (due to oral HPV transmission) [5,10,11]. HPV-positive OPSCC is predominantly associated with HPV16 infection, while a relationship has also been observed with several other high-risk HPV types, such as HPV18, HPV33, and HPV52 [12]. Active HPV infection is accompanied by the expression of the viral E7 oncoprotein, which abrogates pRB functions. The repression of pRB leads to the overexpression of cellular p16INK4A, which is widely being used as a surrogate marker for HPV oncogenic activity [13,14,15]. The addition of p16INK4a (p16) immunohistochemistry staining to the HPV DNA test has been recommended in guidelines to improve the accuracy of HPV diagnosis in HNSCC cases [16]. According to cancer statistics data provided by the Turkish Ministry of Health, on average, 1577 newly diagnosed HNSCC cases were recorded annually between 2013–2017, with no significant rise in this period [17]. In recent years, only a limited number of studies have been conducted in Turkey to address the association of HPV infections with HNSCC in Turkish patients [18,19,20,21,22,23,24]. The overall aims of this report were therefore to determine the prevalence of HPV infections in HNSCC patients and to describe the HPV type distribution among HNSCC cases.

## 2. Results

A total of 106 specimens were obtained from patients whose ages ranged from 19 to 90 years (mean = 59.7 years). Primary tumors were located in the oropharynx (n = 72) or the oral cavity (n = 34). p16INK4A expression was analyzed by performing immunohistochemical staining (IHC). Staining was graded depending on their p16INK4A expression level and pattern. Eighteen HNSCC samples showed p16INK4A overexpression and were considered positive as per the guideline from the College of American Pathologists (CAP) [25]. The p16INK4A evaluations of four samples could not be performed due to quality problems (three HPV DNA positive and one HPV DNA negative). Twenty-six of one hundred and six (24.5%) HNSCC samples were found to be HPV DNA positive. Out of these patients, 22 (84.5%) had tested positive for HPV16, 2 individuals had HPV18, and 1 each patient had HPV45 and HPV82. In OPSCC and oral cavity carcinomas, HPV DNA was detected in 20/72 OPSCC cases (27.8%) and in 6/34 oral cavity carcinomas (17.6%) (Table 1). HPV16 and HPV18 co-infection was detected in one tonsillar sample. HPV16 was detected in the vast majority of samples. HPV18, HPV45, and HPV82 were detected in buccal mucosa and two tonsillar samples. From the various tumor subsites, high prevalence rates of HPV DNA were predominantly detected in the palatine tonsils (Table 2). HPV DNA positivity was found to be significantly higher in individuals under 60 years of age (*p* = 0.024). HPV DNA positivity in tumor samples from non-smokers was significantly higher than in those extracted from smokers (40.6% (13/32) vs. 17.6% (13/74), *p* = 0.011). However, there was no discernible correlation between alcohol consumption and HPV infection. In addition, no relation was found between HPV infection and lymph node status as well as tumor classification (*p* > 0.05).

We calculated the agreement between HPV DNA and p16INK4A expression results, and the tests showed a concordance of 91.2%. The Kappa score was 0.726, indicating substantial agreement to the Landis–Koch reference value.

## 3. Discussion

Common risk factors for HNSCC are alcohol consumption, smoking, being over 60 and living in today’s developed Western countries [8]. In recent years, the rate of HPV-positive HNSCC cases, especially HPV-associated OPSCC, has rapidly increased in the USA [26]. In the 20-year period from 1984 to 2004, the incidence of HPV-positive OPSCC has drastically increased by 225% [27]. In our study, the highest HPV prevalence was found in OPSCC, especially in the palatine tonsils, and HPV DNA was also observed in cancers of the oral cavity. Our observations strongly suggest that there may be an important relationship between HPV infection and HPV-associated carcinogenesis in the oropharynx, especially in the region of the palatine tonsils. The results of our study underpin the results of previous international studies [28] and greatly contribute to the global data on the prevalence of HPV DNA in malignancies in the head and neck region. We did not observe a complete overlap between HPV DNA PCR and p16INK4A immunohistochemical staining results. p16INK4A positivity was observed in 16 of 23 samples (69.5%) with HPV DNA positivity. In real-time quantitative PCR tests that were carried out in the study, there were two p16INK4A positive samples that were negative for HPV DNA. IHC p16INK4A-positivity and HPV DNA positivity correlates significantly in HNSCC. However, there may still be a discrepancy rate of approximately 10 % between these two measurements [29]. To date, it is unclear whether these discrepancies are even real, or if these are artificial discrepancies due to limitations in specificity/sensitivity [30]. Previous studies have reported that individuals with HPV-positive / p16INK4A-negative and HPV-negative / p16INK4A-positive HSNCC show different prognoses and indicated the existence of a p16INK4A-associated HNSCC subgroup independent of HPV status [30]. HPV-positive OPSCC patients in the present study accounted for 40.6% cases among non-smokers, 27.9% among non-alcoholic and 35.7% among 19–60-year-old individuals. Although HPV-associated tumorigenesis seems statistically to be only age-related, HPV-positive HSNCC are more common in patients who do not smoke and do not drink alcohol at young ages, which is in line with previously published studies [31,32]. Previous research has shown that high-risk HPV types dominate in HPV-positive HNSCCs. Baboci et al. [33] found that 95% of HPV-positive HNSCCs carried HPV16 sequences, while the only other HPV type identified was high-risk type was HPV58. Ni et al. [34] demonstrated high-risk HPV types in 26.4% of tumor samples, with the vast majority (71%) being infected with HPV16. Other high-risk types included HPV18, HPV33 and HPV82. In the present study, we predominantly found the high-risk HPV types 16, 18, 45 and 82 in HPV-positive tumors. In contrast, we did not detect any low-risk HPV types in the tumor samples. These results suggest that, among the various HPV types, HPV16 plays a pivotal role in HNSCC development and particularly in OPSCC tumorigenesis. Smoking is a major risk factor for the development of HNSCC. Smoking has decreased in recent years, which is paralleled by a decrease in the incidence of smoking-related diseases in developed countries [35]. Recent studies have shown that the frequency of OPSCC increases among both HPV16-positive and HPV16-negative smokers [36]. In Turkey, 46.1% of the male population uses tobacco, whereas only 15.7% of women are smokers. Moreover, Turkey is one of the countries with the highest mortality rate due to smoking worldwide, with 26.1% of men and 7.6% of women developing fatal conditions due to tobacco abuse [37,38]. Although the vast majority of patients who participated in our study were smokers, the incidence of HPV in non-smoking patients was observed to be higher than those who are light and heavy smokers. Initially, studies on HNSCC in Turkey were conducted as local studies with a low number of patients [18,19]. By performing in situ hybridization against HPV DNA, Umudum et al. (2005) found an HPV prevalence of 15% in HNSCC studied in Ankara [19]. In a previous study conducted in Istanbul on 81 OPSCC biopsies HPV DNA was detected by PCR in 52% and HPV16 was observed in 86% of the positive tested tumors [21]. Since p16INK4A staining was not performed, the real HPV prevalence remains unanswered in these studies. The data from the current study suggest that HPV infection accounts for about 1/4th of the HNSCC cases in patients from Istanbul. According to our data on OPSCC, the frequency of HPV was found to be 27.8%. HPV16 was observed in 85% of the HPV DNA positive cases and is therefore an important etiological agent in OPSCC development in Turkey. 

There are several limitations to the current study. First, we have no data on the sexual behavior of the patients. In addition, since a commercial kit was used in the current study, HPV types that were not included in the kit could not be examined. Finally, our study was conducted using samples from two centers in Istanbul, and the results may therefore not be representative of Turkey.

In a study investigating the frequency of HPV in tonsil tumor-free tissues in children and young adults, the prevalence was detected to be at about 6.3%, indicating that HPV can settle in the mouth at a very early age [39]. Considering that Turkey has a young population who, in general, practices oral sex [40], HPV vaccines known to reduce oral HPV prevalence may represent the most effective tool for the prevention of HPV-induced HNSCC. In Turkey, HPV vaccination is not included in the National immunization programme. The inclusion of HPV vaccines in the national vaccination schedule undoubtedly would not only contribute to the prevention of cervical cancers but also aid in halting the rise in HPV-related HNSCC.

## 4. Materials and Methods

### 4.1. Study Population

This study was approved by the Ethics Committee of the Dentistry Faculty, Istanbul University (Approval Number: 2015/9-6). Informed consent was obtained from all patients who were accepted to be enrolled in the study. This study included a total of 106 patients (78 male/28 female) with a clinical diagnosis of HNSCC. In this study, fresh or frozen biopsies from patients with histologically confirmed HNSCC were collected at the Head and Neck Surgery, Istanbul Faculty of Medicine, as well as the Sisli Hamidiye Etfal Training and Research Hospital, between January 2015 and January 2019. HNSCC biopsies were immediately transferred to and stored at −80 °C in Copan eNAT specimen transport medium (Copan Diagnostics Inc., Brescia, Italy), followed by the extraction of genomic DNA. Viral DNA was isolated from 300 µL of patient oral biopsies using the GeneAll Ribospin vRD Viral RNA/DNA Extraction Kit (GeneAll Biotechnology Co., Seoul, Korea) according to manufacturer’s instructions.

### 4.2. HPV Genotyping and p16INK4a Staining

Anyplex™ II HPV28 kit was performed according to manufacturer’s instructions (Seegene, Seoul, Korea), using 5 µL DNA in each of the two 20 µL reaction mixtures with primer sets A or B, respectively [41]. The Anyplex™ II HPV28 kit semi-quantitatively distinguishes 28 HPV genotypes (namely types 6, 11, 16, 18, 26, 31, 33, 35, 39, 40, 42–45, 51–54, 56–59, 61, 66, 68–70, 73 and 82) in two reactions that can be resolved on the CFX96 real-time PCR device (Bio-Rad, Hercules, CA, USA) simultaneously, thus allowing the identification of the vast majority of clinically relevant HPV types. Cycling conditions encompassed an incubation period of 4 min at 50 °C (to activate the uracil DNA glycosylase in order to prevent contamination), denaturation for 15 min at 95°C, followed by 50 cycles /30 s at 95 °C, 1 min at 60 °C and 30 s at 72 °C. The fluorescence levels were constantly measured as the temperature increased. Melting temperature analysis conditions included cooling to 55 °C, holding at 55 °C for 30 s and heating from 55 °C to 85 °C (5 s/0.5 °C), which was measured after PCR cycles 30, 40 and 50. DNA of the HPV L1 gene and a human housekeeping gene (human beta-globin) were co-amplified simultaneously as an internal control. Data interpretation was performed with the Anyplex software (Seegene, Seoul, South Korea) according to the manufacturer’s instructions. p16INK4A expression was detected on whole tumor sections using the CINtec Histology kit (Roche mtm Laboratories, Mannheim, Germany) according to antibody suppliers’ and standard protocols [42].

### 4.3. Statistics

Statistical analysis was performed using Pearson chi-square and Fisher’s exact test. Differences with a *p* value < 0.05 were considered significant. Concordance between HPV DNA and p16 expression results was assessed by Cohen’s kappa score.

## Figures and Tables

**Table 1:** Demographic variables of the study group.

Characteristic	Total	HPV DNA+ n (%)	HPV DNA− n (%)	*p* Value
Male	78 (73.6)	18 (23.1)	60 (76.9)	0.562
Female	28 (26.4)	8 (28.6)	20 (71.4)	
Age at diagnosis	59.7	55.2	61.15	
Range	19–90	24–70	19–90	
<60	49 (46.2)	17 (34.7)	32 (65.3)	0.024 *
≥60	57 (53.8)	9 (15.8)	48 (84.2)	
Smoking (N) ^a^				
Never	32 (30.2)	13 (40.6)	19 (59.4)	Reference
<400	21(19.8)	3 (14.3)	18 (85.7)	0.041 *
≥400	53 (50)	10 (18.9)	43 (81.1)	0.028 *
Alcohol consumption (N) ^b^				
Never	61 (57.5)	17 (27.9)	44 (72.1)	Reference
Light	13 (12.3)	1 (7.7)	12 (92.3)	0.123
Heavy	32 (30.2)	8 (25)	24 (75)	0.766
Lymph node status (N)				
Negative	47 (44.3)	9 (19.1)	38 (80.9)	0.250
Positive	59 (55.7)	17(28.8)	42(71.2)	
Tumor location (N)				
Oropharynx	72 (67.9)	20 (27.8)	52 (72.2)	0.257
Oral cavity	34 (32.1)	6 (17.6)	28 (82.4)	
Tumor classification (N)				
T1	24 (22.6)	6 (25)	18 (75)	0.490
T2	31 (29.2)	10 (32.3)	21 (67.7)	
T3	29 (27.4)	7 (24.1)	22 (75.9)	
T4	22 (20.8)	3 (13.6)	19 (76.4)	
p16 Immunochemistry ^c^				
Negative	84 (82.4)	7 (8.3)	77 (91.7)	<0.001 *
Positive	18 (17.6)	16 (88.9)	2 (11.1)	

^a^ Brinkman index: daily cigarettes x years. ^b^ Light drinker ≤ 50 g alcohol per day; Heavy drinker > 50 g alcohol per day. ^c^ Four samples (three HPV DNA positive and one HPV DNA negative) were of poor quality for p16INK4A staining. * statistically significant.

**Table 2:** Distribution of HPV types in the various tumor subsite.

Site	Subsite	Specimens	HPV DNA+ (n%)	HPV DNA− (n%)	HPV Types
Oropharynx	Palatine tonsils	38 (35.8)	12 (31.6)	26 (68.4)	16, 18, 82
	Base of tongue	28 (26.4)	5 (17.9)	23 (82.1)	16
	Lateral wall	0	0 (0)	0 (0)	-
	Posterior wall	0	0 (0)	0 (0)	-
	Soft palate	6 (5.7)	3 (50)	3 (50)	16
Oral cavity	Oral tongue	16 (15.1)	1 (6.2)	15 (93.8)	16
	Buccal mucosa-lip	6 (5.7)	4 (66.7)	2 (33.3)	16, 45
	Floor of mouth	7 (6.6)	0 (0)	7 (100)	-
	Hard palate	5 (4.7)	1 (20)	4 (80)	16
Total		106 (100)	26 (24.5)	80(75.5)	
